# Timing-specific parental effects of ocean warming in a coral reef fish

**DOI:** 10.1098/rspb.2023.2207

**Published:** 2024-05-22

**Authors:** L. C. Bonzi, R. K. Spinks, J. M. Donelson, P. L. Munday, T. Ravasi, C. Schunter

**Affiliations:** ^1^ The Swire Institute of Marine Science, School of Biological Sciences, The University of Hong Kong, Hong Kong; ^2^ ARC Centre of Excellence for Coral Reef Studies, James Cook University, Townsville 4810, Australia; ^3^ Blue Carbon Section, Department of Climate Change, Energy, the Environment and Water, Australian Government, Brisbane 4000, Australia; ^4^ College of Science and Engineering, James Cook University, Townsville 4810, Australia; ^5^ Marine Climate Change Unit, Okinawa Institute of Science and Technology Graduate University, Okinawa 904-0495, Japan; ^6^ State Key Laboratory of Marine Pollution and Department of Chemistry, City University of Hong Kong, Hong Kong

**Keywords:** climate change, transgenerational plasticity, non-genetic inheritance, gene expression, transcriptomics

## Abstract

Population and species persistence in a rapidly warming world will be determined by an organism’s ability to acclimate to warmer conditions, especially across generations. There is potential for transgenerational acclimation but the importance of ontogenetic timing in the transmission of environmentally induced parental effects remains mostly unknown. We aimed to disentangle the effects of two critical ontogenetic stages (juvenile development and reproduction) to the new-generation acclimation potential, by exposing the spiny chromis damselfish *Acanthochromis polyacanthus* to simulated ocean warming across two generations. By using hepatic transcriptomics, we discovered that the post-hatching developmental environment of the offspring themselves had little effect on their acclimation potential at 2.5 months of life. Instead, the developmental experience of parents increased regulatory RNA production and protein synthesis, which could improve the offspring’s response to warming. Conversely, parental reproduction and offspring embryogenesis in warmer water elicited stress response mechanisms in the offspring, with suppression of translation and mitochondrial respiration. Mismatches between parental developmental and reproductive temperatures deeply affected offspring gene expression profiles, and detrimental effects were evident when warming occurred both during parents’ development and reproduction. This study reveals that the previous generation’s developmental temperature contributes substantially to thermal acclimation potential during early life; however, exposure at reproduction as well as prolonged heat stress will likely have adverse effects on the species’ persistence.

## 1. Introduction

As a result of anthropogenic climate change, a rise in mean ocean temperatures is happening at an unprecedented rate, and extreme thermal events, such as marine heatwaves, are occurring with increasing frequency and intensity [1,2]. Ocean warming is predicted to impact the distribution and abundance of marine ectothermic organisms [3,4], and tropical species such as coral reef fish might be especially vulnerable to prolonged thermal stress, having evolved in relatively stable thermal environments [5] and living close to their thermal optimum [6]. Accordingly, increases in water temperature have been shown to cause a rise in oxygen demand [7,8], which, if not met, adversely impacts several traits of coral reef fishes, such as respiratory scope [9], swimming ability [10] and reproductive output [11]. However, several fish species have shown the potential to compensate for some detrimental effects of ocean warming through acclimation via phenotypic plasticity [12–16]. Switches in energy production mechanisms and substrates, modifications in the protein synthesis machinery, as well as changes in immune and stress-responsive gene expression were identified among the major molecular processes underlying such plastic accommodations, allowing acclimation to increases in water temperature [17–20]. Ultimately, thermal plasticity may enable populations and species to persist in a rapidly warming world and offer a lifeline for genetic adaptation to catch up over the longer term.

Acclimation to environmental changes via phenotypic plasticity, however, can be restricted to specific ontogenetic windows of increased sensitivity to external conditions [21]. Coral reef fish, as well as other stenothermal species, for instance, can lack the plasticity to acclimate to increases in temperature during adulthood [11,22] while showing acclimation potential when warming is experienced at development [12,14]. Early-life stages can indeed be the most sensitive periods to environmental cues and often show the highest potential for within-generation plasticity (WGP) [23–25], occurring when the organism’s phenotype is affected by its own experience of the environment. Interestingly, the environmental stimuli perceived during development have been found not only to shape the individual’s phenotype but also to affect subsequent generations via transgenerational plasticity (TGP) [25]. Here, we consider TGP in the inclusive sense of when the experience of previous generations (e.g. parents) influences the offspring phenotype (TGP *sensu* Bell & Hellmann [26]), and not just when the parental environment interacts with the offspring’s one (anticipatory TGP or TGP *sensu* Salinas *et al*. [27]). The parental exposure window that is generally thought to have the strongest effect on TGP is during reproduction because of the temporal proximity and, therefore, higher cue reliability between the environmental conditions experienced by parents and the future offspring environment [26,28]. However, gametogenesis, spawning and embryogenesis are also the life stages that are most vulnerable to warming in fish, with negative consequences on egg quality and sperm production [29,30]. Furthermore, in external-fertilizing species, water temperature can also affect gamete performance and molecular composition post-release, with consequences for fertilization success and offspring traits [31]. Therefore, heat stress during reproduction, e.g. because of a heatwave, might adversely affect both parents and their offspring, instead of providing the opportunity for beneficial parental effects.

While beneficial parental effects are at the basis of adaptive TGP and offspring fitness increase in the changed environment, parental effects that are detrimental to the offspring can also occur. Maladaptive anticipatory TGP might arise when the environments of parents and offspring do not match, while negative parental carry-over effects can either result from trade-offs between the costs of survival and growth of the parents against the energy invested in reproduction and offspring fitness, or from simple transmission of poor parental condition [32,33]. Californian mussels (*Mytilus californianus*) offspring of thermally exposed parents show reduced tolerance to warming [34], and coral reef sea urchin (*Echinometra* sp. *A*) parental exposure to future climate conditions negatively affects offspring survival [35]. Therefore, exposure to environmental change across generations does not necessarily lead to acclimation and/or adaptation, and the possibility of decreased offspring fitness and detrimental carry-over effects also exists.

The exposure to environmental changes, such as warming, at different ontogenetic timings and/or in different generations, e.g. because of heatwaves, could have neutral, additive or interactive effects [27,36,37]. In the simplest scenario, a cue elicits the same response regardless of when and how many times it is perceived. In the three-spined stickleback *Gasterosteus aculeatus,* predator-induced WGP and TGP elicit similar phenotypical and molecular responses [38]. Alternatively, the same cue experienced multiple times could reinforce the information, ensuring detection and therefore response (overcoming environmental noise and/or reaching a discrimination threshold) [26], or elicit a stronger, additive response compared to the single experience. In the snail *Nucella lapillus*, when both parents and embryos are exposed to predation risk, offspring size is larger at emergence [39], while additive WGP and TGP effects on growth rate are found in thermally exposed sheepshead minnows (*Cyprinodon variegatus*) [40]. Contrasting cues could also be experienced at different times, raising questions on how different experiences interact and are integrated by the organism. For example, the relative importance of parental development and reproduction—the two most sensitive ontogenetic windows in TGP—is still debated, and while sometimes early-life exposure is needed to induce positive parental effects (e.g. [41]), in other cases reproduction at elevated temperatures is enough to elicit multigenerational thermal TGP (e.g. [42]). On the other hand, especially in organisms where plasticity is only available at development, as in the case of coral reef fishes and thermal plasticity, the stressful exposure to environmental changes during adulthood at reproduction could result in negative parental effects with detrimental consequences on the offspring. Because of such potentially opposite outcomes, it is therefore paramount to explore how environmental cue exposure at different sensitive windows affects TGP. Finally, not all traits have the same plasticity potential, and individual traits might respond differently to the same environmental cue perceived at different exposure timings [43–45]. In *Strongylocentrotus purpuratus* sea urchin, DNA methylation shows TGP in response to simulated upwelling conditions, while WGP alone controls spicule length [46]. Ultimately, the resulting phenotype and acclimation potential of organisms and populations to environmental perturbations will depend on how different traits will be affected and how the environmental cues experienced by parents during the most sensitive windows—development and reproduction—will interact and be integrated with the offspring’s own environmental perception.

Although highly sensitive to warming, the coral reef fish *Acanthochromis polyacanthus* (Bleeker 1855), or spiny chromis damselfish, is able to partially acclimate to warmer temperatures when exposed early in life (developmentally) and to fully restore deficits in aerobic scope when both parents and offspring are exposed to warmer conditions (transgenerationally) [13,17,18]. A shared suite of differentially expressed genes in the liver, mainly related to lipid, protein and carbohydrate metabolism, immune system and transcriptional regulation, has been found to be related to WGP and TGP mechanisms in this species [20]. Previous studies, however, have continuously exposed parents to warming from hatching to breeding, therefore preventing insights into critical thermal windows for parental exposure that induce TGP. For example, to convey beneficial effects on the offspring, parents may only need to experience warming as adults during gametogenesis and reproduction, or alternatively, they may need to be exposed to warmer conditions during development in early life. While exposure timing within the F2 generation was explored by Bernal *et al*. [17], there are some limitations because one of the orthogonal F3 crosses is missing and both F1 and F2 generations were exposed continuously before testing the impacts of reproductive thermal exposure. Therefore, further investigation into the role of warming during parental developmental and reproductive stages is needed to improve our understanding of the interplay between the timing of environmental variation and plasticity. Finally, since reproductive exposure in this species also coincides with embryogenesis because of nest care, the independent exposure of parents to elevated temperature during development or during reproduction alone is necessary to determine whether the transgenerational acclimation effects reported so far are indeed TGP or rather offspring developmental WGP due to embryo exposure to warming.

**Figure 1.**
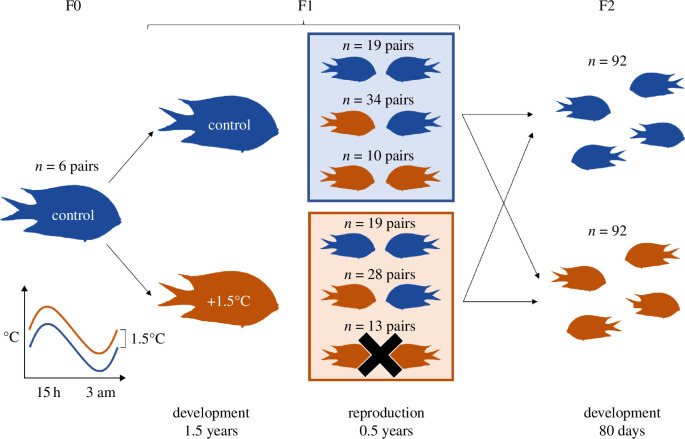
Experimental design. F1 *A. polyacanthus* from wild-caught F0 developed either at present-day control temperature (control—blue) or elevated temperature (+1.5°C—orange) with seasonal and daily fluctuations. At 1.5 years of age, F1 individuals were paired in reciprocal sex crosses of the two thermal treatments and further exposed to control (blue rectangle) or elevated (orange rectangle) reproductive temperatures. The black ‘X’ indicates the F1 treatment that did not reproduce. F2 siblings were split after hatching into control or elevated temperatures, where they developed for 80 days.

In this study, we exposed F1 *A. polyacanthus* to present-day control or +1.5°C average increased temperature during development and at reproduction (figure 1). Breeding pairs were created such that different thermal combinations of sex and time (development and reproduction) occurred. F2 offspring clutches from these parents were split post-hatching into control or +1.5°C, where they developed for 80 days. Elevated temperature affected reproductive success, as well as offspring quality and growth [47,48]. Exposure to warming at reproduction caused low reproductive output and poor hatchling quality [48]. At 3 months post-hatching, offspring from parents that reproduced at elevated temperatures were still smaller and lighter compared to offspring from control parents [47]. Additionally, parental exposure to warming at development also resulted in lower offspring conditions. Parental exposure to elevated temperature, irrespective of ontogenetic timing, seems therefore to be causing maladaptive effects in the offspring. Alternatively, reduced weight might represent a trade-off with the adaptive parental effects on metabolism previously recorded for this species [13,17]. Here, we assessed the genome-wide liver gene expression of the F2 offspring raised at elevated or control temperatures from different parental combinations. The 184 analysed transcriptomes allowed us to explore the molecular responses of *A. polyacanthus* to warming at different ontogenetic timings over two generations and disentangle the effects of the parental experience during development from the reproductive and the offspring developmental exposures. Because of the concurrent increase in mean ocean temperatures as well as more frequent and intense heatwaves, this study aimed to answer unresolved questions related to transgenerational acclimation potential to ocean warming, especially considering the possibility for temperature mismatches between generations and also during an individual’s lifespan.

## 2. Methods

### (a) Experimental design

In order to investigate the importance of exposure timing in the response to warming, we analysed liver gene expression of *A. polyacanthus* exposed to elevated temperature over two generations. Detailed descriptions of the experimental setup are provided by Spinks *et al*. [47,48]. Briefly, adult spiny chromis damselfish (F0 generation) were collected from the wild in the Palm Islands region (18°37′S, 146°30′E) and Bramble Reef (18°22′S, 146°40′E) of the central Great Barrier Reef in Australia, paired and housed with seasonally cycling water temperature resembling the collection site (see electronic supplementary material). During the Austral summer of 2016, F0 pairs bred, and egg clutches were kept with parents until hatching, for them to provide nest care as in the wild. Newly hatched F1 siblings from six breeding pairs were split between a present-day control and an elevated temperature treatment. Larvae were fed live *Artemia nauplii* and then weaned to commercial pellets (see electronic supplementary materials). The water temperature of the two thermal treatments was finely controlled to match simulated seasonal (winter minimum 23.2°C, summer maximum 28.5°C) and diurnal (constant cycling between daily minimum −0.6°C at 3.00 and daily maximum +0.6°C at 15.00) cycles of the Palm Islands for the control treatment, with the elevated thermal treatment equally fluctuating, but 1.5°C higher. This temperature regimen was chosen to match the projections for average ocean temperature increase by 2100 [49] and already occurring heatwaves [50]. F1 fish were kept in the two thermal treatments until maturity (~1.5 years of age), when fish from different families were paired for same and reciprocal sex crosses of the developmental temperatures, resulting in four pair combinations of males and females reared in present-day control and elevated temperatures. The formed pairs were further placed into present-day control or +1.5°C reproductive temperatures, for a total of eight pair combinations (electronic supplementary material, table S1). The F2 generation was produced between December 2017 and April 2018, although no reproduction occurred for the elevated reproductive thermal environment when both parents developed at +1.5°C. Since eggs were kept with their parent until hatching, embryogenesis occurred at reproductive temperature. Within 6 h of hatching, F2 generation siblings from three to five breeding pairs per parental treatment were split into present-day control or +1.5°C thermal treatments (figure 1), which followed the above-mentioned seasonal and diurnal cycles of water temperature. At 80 days post-hatching, F2 fish were sexed by external examination of their urogenital papilla, and two males and two females per clutch per treatment (12–20 individual fish per treatment; electronic supplementary material, table S2) were euthanized by cervical dislocation, measured for standard length, weighed and dissected. Livers were immediately snap-frozen in liquid nitrogen and stored at −80°C for subsequent RNA extraction. The liver was chosen as the target tissue because of its major role in metabolism and to allow comparisons with previous works [17,18,20]. All samples were collected at times between 9 am and 12 pm. The offspring survival rate was measured, and differences between treatments were tested with the Kruskal–Wallis rank-sum test in R (v.3.6.3) [51].

### (b) RNA sequencing

Frozen livers were homogenized in Qiagen RLT Plus buffer for 30 s with single-use silicone beads in an MP Biomedicals FastPrep-24 homogenizer. Total RNA was isolated from whole homogenized livers using a mirVana miRNA Isolation kit, following the manufacturer’s protocol for total RNA isolation procedure. A Nanodrop (Thermo Scientific) and a 2100 Bioanalyzer (Agilent) were used to determine the quantity and quality of the isolated RNA. RNA-Seq libraries were prepared using the Illumina TruSeq stranded mRNA Library Preparation Kit. Library quality check and quantification were performed with a Bioanalyzer High-Sensitivity DNA assay (Agilent). Paired-end fragments of 150 base pairs were sequenced with an Illumina HiSeq 4000 at the King Abdullah University of Science and Technology Bioscience Core Lab. Samples from different thermal treatments were randomly assigned to each lane to avoid batch effects during sequencing, for a total of 184 sequenced samples (electronic supplementary material, table S2).

### (c) Gene expression analyses

Read quality check was performed with FastQC [52] before and after quality trimming and adapter removal by Trimmomatic (v.0.39) [53] with parameters: ‘TRAILING:3 SLIDINGWINDOW:4:15 MINLEN:40’. Trimmed reads were mapped against the *A. polyacanthus* genome (ENSEMBL ASM210954v1) using HiSAT2 (v.2.2.1) [54] with default settings, specifying strand-specific information (--rna-strandness = RF). The featureCounts function from the Subread package (v.2.0.2) [55] was used to calculate gene counts, in read pair counting mode, allowing for multi-mapping fractional computation.

The DESeq2 package (v.1.26.0) [56] was used to statistically analyse differential gene expression in R. The presence of outliers and batch effects in the data was evaluated through clustering and visualization using variance stabilized transformed (VST) counts. Based on principal component analyses and heatmaps of the sample-to-sample distances, six outlier samples were identified and excluded from further analyses (electronic supplementary material, table S2). Likelihood ratio tests (LRTs) were used to determine the best model and get an overview of the overall effects of the different timings of exposure. The chosen design formula included the variable ‘family’ to control for differences due to the parental lineage, and the main effects of parental thermal experience with five levels: (i) both parents raised at control, bred at control–CC, (ii) both parents raised at control, bred at elevated temperature–CH, (iii) one parent raised at control and the other at elevated temperature, bred at control–hC, (iv) one parent raised at control and the other at elevated temperature, bred at elevated temperature–hH, and (v) both parents raised at elevated temperature, bred at control–HC, and offspring post-hatching developmental thermal environment with two levels: (i) control and (ii) elevated temperature. Since no significant interaction was found with offspring temperature, the final model did not include such an interaction term. Differentially expressed genes (DEGs) were statistically determined for the main effects using false discovery rate (FDR) adjusted *p*‐value < 0.01 [57] and a mean expression of  >10 reads (baseMean) as threshold.

Next, the LRT-identified DEGs related to the main effect of parental thermal experience were hierarchically clustered based on expression pattern similarity using the degPatterns function from the DEGreport R package [58] to identify potential interactive effects between the two parental exposure timings. The function was run on the VST processed count matrix of such genes with default settings, except for cluster outlier removal.

To explore the different effects of the three exposure timings, as well as the differences between the developmental exposure of one versus two parents, pairwise comparisons were run (Wald tests) using the same design formula described for the LRT above. DEGs were identified by the following cut-offs: FDR < 0.01, apeglm shrunken |log2 Fold Change (log2FC)| ≥ 0.3 to reduce false positives [59] and baseMean  >10.

Finally, weighted gene correlation network analyses were run with R package WGCNA v.1.70-3 [60] to identify clusters of highly correlated genes and relate them to the experimental treatments. The analysis was run using VST counts of genes with average counts >1 in more than 11 samples, which is the smallest sample set per treatment. A soft-thresholding power *β* of 7 was chosen based on network topology analysis, and gene network clusters were identified using the automatic one-step network construction and module detection blockwiseModules function, using a signed topological overlap matrix (TOM), a minimum module size of 30 and a threshold of 0.3 for merging modules. Gene modules were then correlated with the parental developmental, reproductive and offspring developmental thermal treatments, to identify which gene clusters were significantly associated with each different thermal exposure timing (*p*‐value < 0.001). A module–trait relationships heatmap was produced and modules with significant correlations were further investigated.

Functional enrichment analyses of DEGs identified by DESeq2, as well as gene sets belonging to degPatterns clusters and significant modules detected by WGCNA were performed in OmicsBox (v2.0.36) [61] with Fisher’s exact test (FDR < 0.05).

## 3. Results

**Figure 2.**
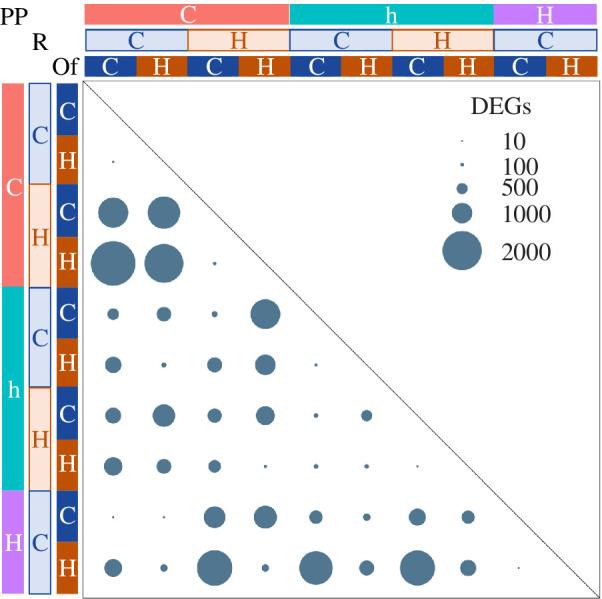
Offspring DEGs due to different thermal treatments. DEGs from pairwise comparisons between offspring with contrasting thermal histories. ‘PP’ stands for parental pair developmental thermal condition, where ‘C’ = both parents developed at control temperature, ‘h’ = one parent developed at control and one at +1.5°C and ‘H’ = both parents developed at +1.5°C. ‘R’ stands for reproductive and ‘Of’ for offspring developmental thermal conditions, where ‘C’ = control, ‘H’ = +1.5°C. The sizes of the circles are proportional to the number of DEGs between comparisons (FDR < 0.01).

Exposure of *A. polyacanthus* to elevated water temperature at different ontogenetic times greatly affected the 80-day-old juvenile hepatic gene expression profiles. The strongest driver of gene expression variation in the offspring livers was the parental thermal experience (6210 DEGs; electronic supplementary material, figure S1; electronic supplementary material, table S3). Conversely, the offspring post-hatching developmental temperature had the smallest influence on their own expression profile (1483 DEGs; electronic supplementary material, table S4). Accordingly, the largest differences in gene expression were found between offspring with different parental developmental and/or reproductive thermal experiences, while comparing between offsprings raised at different temperatures usually returned the smallest DEG numbers (figure 2). Seven hundred and eighty-five genes (electronic supplementary material, table S5) were influenced by elevated temperature irrespective of the exposure timing (parental or offspring lifetime), and they were mostly involved in metabolism and oxidoreductase activity, including the cytochrome P450 (CYP) superfamily of enzymes.

During rearing, approximately 10% (9.7 ± 1.6 s.e.m.) natural mortality occurred (electronic supplementary material, table S6). While a potential genetic component to the measured responses cannot be excluded, the mortality rate was relatively low and uniform across the treatments (Kruskal–Wallis chi-squared = 7.569, df = 9, *p*-value > 0.05) and six different F0 pairs were used to minimize potential genetic effects. Therefore, we believe that the differences between treatments are due to the parental thermal experiences, rather than to selective forces for temperature tolerance.

### (a) Effects of warming during offspring development

The temperature experienced by offspring at development explained changes in expression levels of 698 genes, which were not altered due to the parental thermal experience (electronic supplementary material, table S4) and were mainly involved in DNA replication and tRNA aminoacylation for protein translation (electronic supplementary material, table S7). When offspring were exposed to elevated temperature, regardless of their parental developmental and reproductive temperatures, genes encoding for components of the MCM complex involved in DNA replication initiation were downregulated, as well as CYP coding genes involved in oxidoreductase activity (electronic supplementary material, table S8). Accordingly, genes involved in DNA replication initiation and DNA repair belonged to a gene network cluster significantly negatively correlated with offspring developmental temperature (64 genes; *p*-value = 6e^–06^; electronic supplementary material, figures S2 and S3; electronic supplementary material, table S9). No interaction between the offspring’s developmental thermal environment and the parental thermal experience was found, therefore offspring response to elevated temperature did not vary significantly if matched or mismatched in temperature with the parental and/or their own embryonic thermal environments.

### (b) Effects of warming during parental development

To explore the effects of parental developmental timing of exposure to warming, we compared offspring of parents that both developed at elevated temperature and reproduced at control to offspring from control parents, regardless of their own developmental temperature. Several RNA polymerase I and III subunits and genes with rRNA- and tRNA-processing functions were upregulated (electronic supplementary material, tables S10 and S11). The downregulated genes showed functions related to vitamin B6 binding, including genes involved in glucose/energy metabolism such as glutamic–pyruvic transaminase (GPT), glutamic–oxaloacetic transaminase 1 (GOT1) and glycogen phosphorylase L (PYGL), as well as steroid hormone-mediated signalling, due to several nuclear receptors (e.g. the subfamily 1 group D member 2—NR1D2, group I member 2—NR1I2, and group F member 6—NR2F6, and RAR-related orphan receptor A—RORA; electronic supplementary material, tables S10 and S11). If only one of the parents developed at elevated temperature, however, their offspring downregulated genes encoding for structural constituents of ribosomes, involved in translation and peptide biosynthesis, while among the upregulated genes, we found genes encoding for endoplasmic reticulum (ER) proteins such as calreticulin (CALR) and torsin family 1 member A (TOR1A), involved in quality control of protein folding, as well as genes with lipid metabolic functions (electronic supplementary material, tables S12 and S13).

### (c) Effects of warming during reproduction

Reproduction at elevated temperature caused downregulation of structural constituents of ribosomes and genes with peptide synthesis function, as well as oxidoreductase activity, ATP synthesis and electron transfer (e.g. cytochrome-c oxidase and NADH:ubiquinone oxidoreductase subunits), regardless of either parental or offspring developmental temperature (electronic supplementary material, tables S14–17). Accordingly, genes coding for structural constituents of ribosomes were found in a gene network cluster significantly negatively correlated with reproductive temperature (*p*-value = 2e^−12^; 741 genes; electronic supplementary material, figure S4*a*; electronic supplementary material, table S18), while genes related to electron transfer activity, cellular respiration, proteasome complex and mRNA splicing were part of a larger cluster of 2161 genes also negatively correlated with reproductive temperature (*p*-value = 2e^–04^, electronic supplementary material, figure S4*b*; electronic supplementary material, table S19). Differences were instead found in the upregulated functions depending on whether any of the parents were also exposed to elevated temperature during development. If both parents were exposed to warming at reproduction only, their offspring upregulated genes related to translation initiation and regulation (e.g. translation initiation factor 2 alpha kinases 2 and 3—EIF2AK2, EIF2AK3—and 4E binding protein 2—EIF4EBP2), protein folding (e.g. TOR1A) and negative regulation of gene expression. Moreover, many of these genes encoded for proteins localized in the ER, e.g. genes in the calnexin/calreticulin (Cnx/Crt) cycle, like CALR, calnexin (CANX), protein disulphide isomerase family A members 3 and 4 (PDIA3, PDIA4) and in the ubiquitin-dependent ERAD pathway, as well as unfolded protein response (UPR) pathway components (ER oxidoreductase 1 alpha—ERO1A, DNAJ heat shock protein family B11—DNAJB11, zinc finger and BTB domain containing 17—ZBTB17; electronic supplementary material, tables S14 and S15). Genes located in the ER and involved in protein folding were also found in a small module positively correlated with reproductive thermal treatment (74 genes; *p*-value = 6e^−05^; electronic supplementary material, figure S4*c*; electronic supplementary material, table S20). On the other hand, if one of the parents not only reproduced but also developed at elevated temperature, their offspring overexpressed genes involved in protein transport, regulation of systemic arterial blood pressure by circulatory renin–angiotensin (angiotensin-converting enzyme 2—ACE2, glutamyl aminopeptidase—ENPEP), protein ubiquitination and apoptosis (electronic supplementary material, tables S16 and 17).

### (d) Parental development and reproduction interactive effects

**Figure 3.**
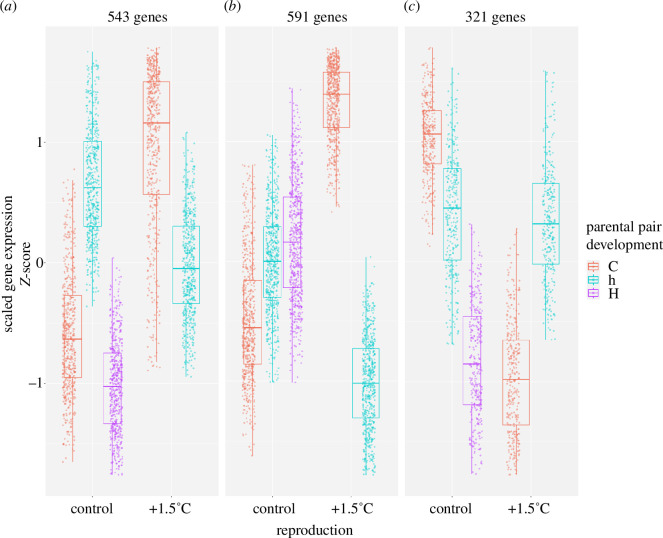
Expression profiles of DEGs showing interactive effects between the two parental exposure timings. DEGs were clustered based on their scaled expression profiles (*Z*-score). The numbers of genes per cluster are provided above each plot. In the parental pair development, ‘C’ = both parents developed at control temperature, ‘h’ = one parent developed at control and one at +1.5°C and ‘H’ = both parents developed at +1.5°C.

The differences in responses to reproduction at elevated temperatures between offspring whose parents did or did not experience warming during development as well suggest the presence of interactive effects between the two exposure timings. Indeed, among the 6210 DEGs due to the parental experience (electronic supplementary material, figure S5), several clusters of genes exhibit different expression levels depending on whether warming was only experienced by parents at development, at reproduction, or by any of the parents at both life stages (figure 3). Genes involved in cell redox homeostasis, carbohydrate metabolism and protein folding quality control in the ER, such as components of the UPR pathway (DNAJB11 and ZBTB17) and members of the Cnx/Crt cycle (CALR, CALX, PDIA3 and PDIA4), belong to a cluster of 543 genes that are overexpressed when parents experienced warming at reproduction, but closer to control levels if any of the parents also developed at elevated temperature, regardless of the offspring developmental temperature (figure 3*a*; electronic supplementary material, table S21). A different cluster (591 genes), similarly upregulated in offspring whose parents experienced warming at reproduction only, also comprises genes involved in protein folding in the ER, such as heat shock protein family A member 5 (HSPA5), UDP-glucose glycoprotein glucosyltransferase 1 and 2 (UGGT1 and UGGT2) and protein kinase C substrate 80 K-H (PRKCSH; figure 3*b*; electronic supplementary material, table S22). Proteasome assembly, RNA processing and DNA damage response are additional processes enriched in this cluster. Finally, genes involved in steroid hormone-mediated signalling belong to a cluster of 321 genes that are strongly downregulated when parents are exposed to warming at reproduction alone, but that show almost no change if elevated temperature is also experienced during parental development (figure 3*c*; electronic supplementary material, table S23).

## 4. Discussion

In this study, we show the conserved and timing-specific molecular signatures of exposure to near-future predicted water temperatures across two generations and reveal that the parental thermal experience has a much greater effect on the offspring transcriptional response at 80 days old than does their own post-hatching developmental experience. Accordingly, in this experiment, offspring length and weight ratio was controlled by the parental thermal exposure [47]. This builds our understanding on the influence of environmental conditions across generations and supports the expectation that parental influence is likely to be strongest in early life [62], while current environmental conditions become the main driver later in development. Parental exposure always affected the transcriptional response of offspring, no matter whether the parents experienced warming only at development, only at reproduction or throughout their life. However, the timing of parental exposure mattered, dictating distinct gene expression changes, with likely contrasting acclimation outcomes for the new generation. Because the consistency between parental and offspring thermal exposures did not affect offspring conditions [47] or molecular responses, these results suggest carry-over rather than anticipatory parental effects [33,45]. On the contrary, mismatches between the temperatures experienced by the two parents at development or across parental lifespan played an important role in shaping offspring thermal acclimation.

The exposure of either parents or offspring to higher temperatures caused the activation of some common molecular responses. In particular, we found modifications in metabolism and energy production, a common response to warming in ectotherms such as fishes, both within [20,63,64] and across generations [17,20]. Moreover, members of the cytochrome P450 superfamily of enzymes, characterized by oxidoreductase activity, were downregulated whenever offspring or their parents were exposed to elevated temperature, which is a possible indication of liver inflammation [65,66] in offspring directly or transgenerationally exposed to warming. The exposure of parents to elevated temperature, however, mostly caused the activation of distinct gene sets compared to the direct exposure of the offspring themselves, similar to the discordant within- and across-generation transcriptional responses of *Daphnia ambigua* in a predator-induced phenotypic plasticity experiment [67]. Exclusive to the offspring’s developmental warming experience was the inhibition of DNA replication and the activation of DNA repair mechanisms. Suppression of DNA replication because of developmental warming might be a sign of the activation of the DNA-replication stress–response pathway [68] to allow time to repair damaged DNA following temperature stress. Alternatively, DNA replication downregulation could indicate energy investment shifts, in agreement with findings from Bernal *et al*. [18], where *A. polyacanthus* offspring downregulated genes involved in DNA replication concurrently with heightened routine oxygen consumption when developmentally exposed to an increase in water temperature compared to the previous generation. By contrast, parental thermal exposure affected processes related to RNA processing and protein synthesis and metabolism, indicators of changes in protein turnover, possibly a conserved transgenerational response to warming found in *A. polyacanthus* as well as in sticklebacks [19,20]. Hence, a combination of shared and distinct mechanisms underlying inter-generational and developmental thermal plasticity is likely in place, revealing a core response to warming but also a decoupling in the two exposure timing effects that could potentially be independently subject to evolutionary pressure [69].

The two different parental exposure timings, either throughout juvenile development until maturity or during reproduction only, also elicited distinct molecular responses in the offspring. Likewise, in sticklebacks, grandparental reproductive and parental developmental exposures elicit different physiological responses in subsequent generations [15,19,70]. Here, the parental developmental exposure to elevated temperature caused changes in offspring energy utilization through downregulation of genes involved in glucose metabolism and nuclear hormone receptors like NR1D2 and RORA, key regulators of the circadian clock and many metabolic functions [71,72]. Parental developmental warming also increased transcription and modification potential of non-coding RNAs, in particular rRNAs and tRNAs, which may indicate heightened protein synthesis as well as cell growth [73,74]. Interestingly, sticklebacks born from mothers developmentally exposed to warming revealed enhanced protein synthesis resulting in higher respiration rates, and, consequently, in the ability to meet the increased oxygen demand in warmer water and maintain aerobic scope [19]. Because *A. polyacanthus* has been similarly found to retain aerobic capacity when transgenerationally exposed to warming [13], our results suggest that increased protein synthesis is a conserved molecular mechanism underlying the acclimation ability in this and other species. Moreover, our findings reveal that such beneficial traits are due to the developmental exposure of parents to increased water temperatures, therefore being adaptive transgenerational responses rather than WGP due to embryo exposure to warming. These positive parental effects were independent of the thermal environment experienced by the offspring themselves, likely representing carry-over effects of an improved parental condition [33] acquired during early-life stages. Elevated temperature exposure at development in *A. polyacanthus*, for example, has been shown to improve key metabolic attributes, such as lowering resting metabolic rates, therefore potentially resulting in these fish having extra energy available for activities like feeding and reproduction [12]. These metabolic adjustments acquired through developmental plasticity might then be passed on to the next generation, resulting in adaptive transgenerational effects, which were also evident in the offspring’s ability to maintain swimming performance [75], despite being lighter and in lower body condition compared to offspring from control parents [47]. Trade-offs seem therefore to be in place between benefits and costs of the metabolic adjustments needed to acclimate to elevated temperature. Nevertheless, our findings indicate how early-life environmental experiences matter in TGP, and parental exposure of *A. polyacanthus* to warming during development appears to be overall beneficial for the offspring and might lead to improved acclimation to increased water temperature.

The benefits of parental development on offspring acclimation potential seemed however to be reduced when only one parent was exposed to elevated temperature during development, while the other developed at control. Indeed, offspring from developmentally mismatched parents exhibited signs of metabolic stress, impairment of the translational machinery and maladaptive swimming speed [75]. Our findings therefore suggest that the adaptive nature of the transgenerational effects due to the developmental exposure timing might depend on the consistency between parental thermal experiences during early life. Moreover, while here we cannot disentangle the individual effects of each parent, paternal and maternal contributions to the offspring acclimation potential might also differ, and future research focused on the individual parental contributions is needed to fully disentangle each parent’s influence on the new generation’s persistence at elevated temperature.

While parental developmental thermal exposure caused trade-offs between costs and benefits of thermal acclimation, reproduction in warmer water was always detrimental. The exposure to elevated temperature during reproduction, which in our experiment also coincided with embryogenesis, indeed caused a marked reduction in the expression of genes involved in protein synthesis and mitochondrial ATP production in the offspring. Similar effects were found in lake sturgeons, *Acipenser fulvescens*, exposed to increased water temperature during early development, a critical life stage in this species [76]. In our study, translation and cellular respiration suppression were accompanied by upregulation of genes indicating heat-induced ER stress owing to the accumulation of unfolded or misfolded proteins in the ER. Genes involved in the calnexin/calreticulin (Cnx/Crt) cycle for protein folding quality control, together with key components of the ER-associated degradation pathway, were upregulated in offspring when reproduction occurred at elevated temperature. This indicates increased amounts of misfolded proteins in the ER, similar to findings in rainbow trout *Oncorhynchus mykiss* kidney following heat stress [77]. Additionally, components of the unfolded protein response (UPR) pathway, activated to re-establish homeostasis when the ER folding capacity is overwhelmed, were upregulated in offspring because of reproduction at elevated temperature. One of these genes, for example, is the eukaryotic translation initiation factor 2α kinase 3 (EIF2AK3), a key stress sensor able to activate UPR and inhibit ribosome assembly and protein synthesis, leading to translation inhibition [78]. Upregulation of UPR markers and liver tissue damage was also found in largemouth bass *Micropterus salmoides* when acutely exposed to elevated temperature [79], while in mice livers ER stress markers and Cnx/Crt genes upregulation persisted for 21 days after a thermal injury, indicating long-term functional alterations of hepatic functions [80]. Such prolonged suppression of protein synthesis and ATP production will likely result in energy limitations, fitness decrease and suboptimal growth [81]. Indeed, when reproduction occurred at elevated temperature, offspring were smaller at hatching and in worse body condition 3 months post-hatching compared with offspring from control parents [47]. This could, therefore, be linked to metabolic dysfunctions, suggesting either negative parental carry-over condition effects or detrimental effects of warming on embryogenesis.

Because of *A. polyacanthus* nest care, not only gametogenesis and spawning but also embryogenesis occurred at the same temperature as parental reproduction. Therefore, the transcriptional patterns caused by the reproductive timing may be due to post-release gamete effects and/or by embryonic WGP, rather than—or in combination with—reproductive TGP. Gametes are vulnerable to environmental temperature, with consequences for offspring traits—especially in externally fertilizing species—such as reduced size and swimming performance in lavaret (*Coregonus lavaretus*) larvae sired by sperms exposed to warming [82]. Environmental cues experienced during embryogenesis are also expected to greatly, and often irreversibly, affect the phenotype through developmental WGP [25], with warming during embryogenesis impacting later fish life traits and transcriptional signatures [83–85]. However, embryonic WGP usually shows adaptive rather than maladaptive consequences, with fish performing better in the warmer environment if exposed to elevated temperatures during embryogenesis [83,84]. The negative effects of warming during reproduction that we measured, therefore, suggest that embryonic WGP may not be the prevalent mechanism at play here. Such negative effects might rather manifest because of post-release gamete exposure or because of reproductive TGP if stressful conditions experienced at reproduction affected parental investment and quality of the resources passed on to the new generation. For example, warming impairs oestrogen synthesis in fish, with negative effects on ovarian function, hepatic vitellogenesis synthesis and oocyte development [86], and spermatogenesis is reduced at elevated temperatures in *A. polyacanthus* [11]. Future work employing experimental designs able to separate gametogenesis, post-release gamete and embryonic WGP effects will help in pinpointing the exact mechanism responsible for the offspring responses to reproductive warming exposure. Nevertheless, this study shows how exposure to elevated temperature at reproduction, e.g. during a heatwave, has very different effects on offspring compared with parental developmental exposure, causing long-term hepatic ER stress in the spiny damselfish, with lasting impairment of the translational and respiratory machineries.

In addition to the individual effects of the two parental exposure timings, the expression patterns of several hundred genes showed interactive effects between parental developmental and reproductive experiences. Such interaction was also found to affect routine oxygen consumption and body condition differently in third-generation sub-adult *A. polyacanthus* [17]. Here, depending on the combination of developmental and reproductive experiences, many genes showed variable differential expression, including the upregulation of genes involved in ER stress, Cnx/Crt cycle and UPR pathway when warming occurred during reproduction and none of the parents developed at elevated temperature. However, as such signals were absent when elevated temperature was experienced by one of the two parents during both parental development and reproduction, some of the detrimental effects of warming seem to be lessened by parental exposure to elevated temperatures during development. This is in line with the hypothesis that temporal autocorrelation between perceived environmental stimuli during successive sensitive ontogenetic windows might work as a positive feedback to reinforce the level of predictability of the future environment, ultimately affecting the reliability of the transmitted information and the adaptive nature of TGP [26,37,87]. Despite some traits showing thermal acclimation when both parental development and reproduction occurred at elevated temperature, exposure to warming throughout the parent lifespan seemed nevertheless to have detrimental effects overall . These offspring still showed suppression of translation and mitochondrial respiration, while also overexpressing genes involved in apoptosis and protein ubiquitination, indicating increased protein degradation. Accordingly, reproduction did not occur when both parents experienced warming throughout their lives. Our results therefore indicate that *A. polyacanthus*, and perhaps other coral reef fishes, will struggle with the predicted increase in water temperature, with potentially serious adverse effects on population viability and persistence.

Persistence of organisms in a warming world will depend on their ability to acclimate, within and across generations, to the changing environment. In this study, we tackled the fundamental question of the importance of ontogenetic timing in the transmission of parental effects. We demonstrated that while a parental developmental experience of warming in *A. polyacanthus* might contribute to adaptive, although not anticipatory, TGP in their juvenile offspring, reproduction in warmer water will not have the same beneficial effects. Rather, if a heatwave should occur during the reproductive season, offspring may suffer from detrimental metabolic effects. Moreover, our results suggest that parental pre-exposure to warming during development alters and potentially worsens the reproductive thermal signature. Finally, we show molecular evidence of physiological stress in offspring of parents with mismatching thermal experiences, suggesting that similar parental thermal histories are important for their offspring's acclimation potential. These results are particularly relevant considering the increasing frequency in extreme thermal events, which will additionally reduce the predictability of the thermal environment, a fundamental aspect in TGP [87,88]. Overall, our results unveil new molecular mechanisms involved in transgenerational response to warming and demonstrate the importance of exposure timing of the previous generation’s environmental experiences for the individuals' capacity to cope with warmer ocean temperatures.

## Data Availability

RNA-seq data can be found under the BioProject PRJNA998209. Electronic supplementary material is available online [89].
